# Predicting Dimensions in Microfluidic Paper Based Analytical Devices

**DOI:** 10.3390/s21010101

**Published:** 2020-12-26

**Authors:** Raquel Catalan-Carrio, Tugce Akyazi, Lourdes Basabe-Desmonts, Fernando Benito-Lopez

**Affiliations:** 1 Microfluidics Cluster UPV/EHU, Analytical Microsystems & Materials for Lab-on-a-Chip (AMMa-LOAC) Group, Analytical Chemistry Department, University of the Basque Country UPV/EHU, 01006 Vitoria-Gasteiz, Spain; raquel.catalan@ehu.eus (R.C.-C.); tugce.akyazi@ehu.eus (T.A.); 2 Microfluidics Cluster UPV/EHU, BIOMICs Microfluidics Group, Lascaray Research Center, University of the Basque Country UPV/EHU, 01006 Vitoria-Gasteiz, Spain; 3 Basque Foundation for Science, IKERBASQUE, 48013 Bilbao, Spain; 4 Bioaraba Health Research Institute, Microfluidics Cluster UPV/EHU, 01006 Vitoria-Gasteiz, Spain; 5 BCMaterials, Basque Centre for Materials, Micro and Nanodevices, UPV/EHU Science Park, 48940 Leioa, Spain

**Keywords:** LOC, wax printing, paper microfluidics, µPAD, paper microfluidics fabrication

## Abstract

The main problem for the expansion of the use of microfluidic paper-based analytical devices and, thus, their mass production is their inherent lack of fluid flow control due to its uncontrolled fabrication protocols. To address this issue, the first step is the generation of uniform and reliable microfluidic channels. The most common paper microfluidic fabrication method is wax printing, which consists of two parts, printing and heating, where heating is a critical step for the fabrication of reproducible device dimensions. In order to bring paper-based devices to success, it is essential to optimize the fabrication process in order to always get a reproducible device. Therefore, the optimization of the heating process and the analysis of the parameters that could affect the final dimensions of the device, such as its shape, the width of the wax barrier and the internal area of the device, were performed. Moreover, we present a method to predict reproducible devices with controlled working areas in a simple manner.

## 1. Introduction

In recent years, paper has gained considerable attention as a substrate material for microfluidic devices thanks to not only its remarkable low cost and universal presence but, also, its mechanical properties, enabling the ease of fabrication/operation, lightness, flexibility and low thickness, as well as biocompatibility and biodegradability [1,2]. Considering the outstanding properties of paper materials, microfluidic paper-based analytical devices (µPADs) represent an innovative, equipment-independent [3] platform technology for fluid analysis, which is generally used for biological [4], environmental [1,5,6,7] and health applications [2,8,9,10]. µPADs enable microfluidic manipulations like transportation, mixing or separation within one analytical run, while allowing the analysis of complex and small amounts of biochemical samples (10^−9^ to 10^−18^ L) [11].

In 1949, a paraffin-patterned paper-based assay was reported as the first fabricated fluidic channel on paper by Muller et al. [12]. Years later, Whitesides’ group at Harvard University presented a protein–glucose assay fabricated by a lithography method in paper, which is considered as the real introduction of paper-based microfluidics into the world, since it marked the development of this technology [13]. Since the main objective of microfluidics is to obtain inexpensive, portable and affordable devices for point-of-care diagnostics and field testing, it is understandable that their fabrication techniques should be simple, easy and cost-effective while compatible with large-scale manufacturing. The aim of any µPAD fabrication protocol should, in principle, meet the specifications required by the WHO for this purpose, the ASSURED criteria, which is an Affordable, Sensitive, Specific, User-friendly, Rapid, Equipment free and Delivered (small and portable) device [14].

As it has been widely reviewed [15,16], many techniques have been used to create hydrophobic barriers in order to contain a hydrophobic sample for the fabrication of paper-based microfluidic devices. Some of them make a physical blocking of the pores of the paper (photolithography, plotting or laser etching), while others make a modification of its surface by chemical treatments (plasma treatment or ink jetting) or by physical deposition of reagents, mainly wax (wax and ink jetting or screen-printing). Nevertheless, not all these techniques fulfill the low cost requirements, e.g., photolithography [17,18,19], stamping methods [20,21,22,23] or plasma deposition, while others are not suitable for mass production, e.g., cut plotting [24,25]. Therefore, the most commonly used techniques are those based on wax, which are, in general, low-cost methods that use nontoxic patterning reagents [26]. Printing methods using either ink [27,28] or wax printers [16,29] are preferred for the fabrication of paper-based microfluidic devices [29,30]. Recently, another wax-based technique has been using pens for the manual deposition of wax; nevertheless, this method is poorly reproducible and scalable. Wax printing, which was introduced by Lu et al. [31] in 2009, despite being a two-step fabrication process, offers an easy, efficient and economic fabrication method potentially scalable for mass production.

On the other hand, flow control in paper microfluidics is still challenging, being nowadays a hot topic in research [32,33,34]. In general, liquid moves thanks to the inherent capillarity properties of paper. This characteristic can be its worst disadvantage, since it does not allow to control the speed and the direction of the flow. This generally leads to problems such as analyte losses during transport, and thus, higher limits of detection than traditional techniques are obtained [35]. Hence, flow control is a determining feature in the functionality of devices for applications like reagent or sample additions and sequential injections or dilutions [29]. In order to solve this critical problem, many mechanisms such as switches, valves and fluidic timers are emerging [36]. For example, Li et al. [37] developed a simple and effective method for selective dosing and flow control, the first microfluidic switch in a µPAD, which allowed or restricted the capillary flow by just applying manual pressure. Another similar method for a programmed sample delivery was reported by Shin et al. [38]. In the pressed region of the device, fluidic delays were created by decreasing and increasing the permeability and fluid resistance, respectively. Lutz et al. [39] introduced paper-based valves obtained by 2D microfluidic networks with different lengths, enabling programmable fluidic disconnects. Despite controlling the sequence of fluid delivery, the mechanism was not sufficient to control the exact delivery time. As another fluid control method, Toley et al. [40] used a tuneable absorbent pad-based shunt placed on top of the main channel to generate a flow delay.

Houghtaling et al. [41] demonstrated a novel dissolvable bridge structure in paper as a shut-off valve for the autonomous delivery of multiple volumes to different channels. Similarly, Lutz and his team presented dissolvable sugar fluidic restrictors to produce flow delays and, thus, programme multistep assays for paper diagnostics [42]. Jahanshahi-Anbuhi et al. [43] used a rapidly dissolving polymer to generate a regulated time flow shut-off valve system. Our team achieved fluid flow manipulation in paper by introducing two different set of ionogel materials as passive pumps [3,44]. Niedl [45] and Wei [46] used hydrogels as fluid reservoirs and flow regulators. More recently, Chatterjee and co-workers [47] designed configurations to control the flow in three-dimensional porous sheets, allowing the movement of liquid by gradients in Laplace pressure, eliminating the pumping requirement. In general, all these reported fluidic control solutions, although highly innovative, include complicated fabrication and operation protocols, which are unacceptable for their use in resource-limited countries, according to the WHO requirements [48,49].

The design of paper-based devices is generally made by ordinary designing software, such as AutoCAD or Adobe Illustrator. However, specific software has been developed for the selection of the shape, width of the barrier and width of the channel [14,50,51]. In particular, during the patterning process of the paper substrate, it is important to be able to obtain the desired dimensions of the wax barriers with well-controlled and uniform features in the final printed and heated device. Nevertheless, it is difficult to predict the final features of the device from the dimensions chosen during the design. The printing and subsequent heating of the design vary the final device shape and dimensions. Well-defined, stable and homogeneous dimensions in the microfluidic channels are the first conditions that enable fluid flow control in µPADs. Only the combination of reproducible fabrication protocols that ensure predictable microchannel features and fluidic elements such as the ones described above will generate µPADs with effective fluid handling and flow control capabilities. Moreover, this extra control during the fabrication process will permit the mass fabrication and industrial scalability of µPADs.

In this paper, we looked through the wax printing process conditions (printing and heating), as well as the parameters that affect the final dimensions of printed devices, to fabricate µPADs with effective fluid handling and flow control capabilities. The results allow us to predict the dimensions of the final device from the design and set the boundaries needed for the fabrication of reproducible µPADs.

## 2. Materials and Methods

### 2.1. Materials

Whatman Filter paper with Grade 1 (Scharlab, Spain) was used as a substrate. For the wax printing method, a wax printer XEROX ColorQube 8580, a hot plate (Labnet International Inc., Mayfield Ave Edison, NJ, USA) and the software design application AutoCAD^TM^ were used. The scanning facility of an HP Laserjet PRO 400, and the Adobe Reader’s measuring tool, were used to obtain the dimensions of the devices.

### 2.2. Fabrication of µPADs by Wax Printing

#### 2.2.1. Heating Process Optimization

Five designs were drawn by AutoCAD and printed on one side of the paper substrate with the wax printer (Table 1). Then, the printed devices were heated with a hot plate at 110 °C and at 125 °C.The width of the channel, both at the front and at the back sides, were measured at 2.5, 3, 4, 5, 6, 7, 8 and 9 min.

Before carrying out the heating process, the temperature of the surface of the hotplate was characterized with a thermometer. The central area of the hotplate showed a homogeneous temperature during heating. In addition, the devices were covered with a flat glass slide to flatten the paper devices and, thus, obtain uniform wax shapes on both sides of the paper.

#### 2.2.2. Wax Barrier and Device Dimension Optimization

The wax barriers and the device dimensions were systematically varied, using simpler structures such as squares, rectangles and circles to obtain the parameters that influence the final dimensions of the devices after the printing and heating processes. The shape, the width of the drawn wax barrier and the size of the device’s internal area were investigated; see Table 2.

## 3. Results and Discussion

The fabrication of the µPADs by the wax printing method consists of three steps: the design of the device, the printing of the device in the filter paper using the wax printer and the heating process. After the design and the wax printing steps—see the Experimental section—the µPADs are heated to define their final dimensions, borders and channels. Therefore, the first parameter to be investigated is the wax barrier that defines the channel width of the µPADs after the heating process both at the top and at the bottom sides of the paper. Full wax penetration into the back side of the paper is necessary to obtain the hydrophobic pattern and, thus, an operative device. Furthermore, the dimensions of the wax barriers on both sides of the paper should be homogeneous in order to ensure a uniform fluid flow in all printed devices. For that reason, the heating temperature step was investigated.

The best heating performance was obtained at a temperature range of 110–125 °C. For lower temperatures, wax diffusion and penetration through the paper substrate was found to be extremely slow and not sufficient to generate homogeneous features at the backside of the paper substrate, even after long periods (>10 min). On the other hand, at temperatures above 125 °C, the paper substrate suffered degradation over time. Zhong et al. found out that, for temperatures above 150 °C, the paper curled up, and the color of the paper changed [52]. Additionally, the barriers were not fully homogeneous from device to device, obtaining that more than 60% of the devices were not operative.

The temperature range of 110–125 °C was investigated, and it was found that the temperature affects the fabrication time but not the shape and operability of the fabricated devices. Table 3 shows the time needed to obtain successful µPADs at 110 and 125 °C using the device configurations (devices-1–5 from Table 1). We concluded that lower temperatures need longer heating times to reach the same device configuration.

**Figure 1:**
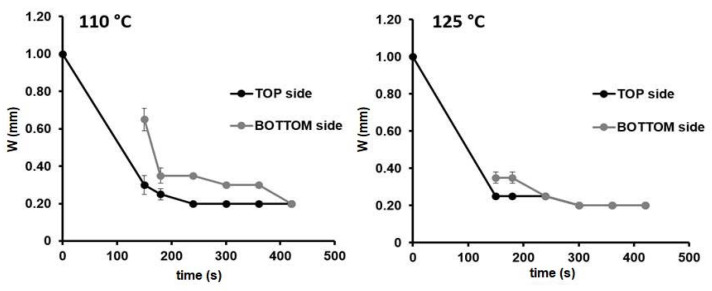
Width changes over time in the front (black) and back (grey) sides of the paper substrate for the microfluidic paper-based analytical device (µPAD) configuration: device-5 (*W_c_* = 1.00 mm) during the heating process at 110 and at 125 °C, n = 3.

For instance, considering the device5 design (see Figure 1), the two temperatures generated the same channel width in both sides of the paper substrate (0.20 mm). The only difference was the time needed to reach the final device configuration of 420 s at 110 °C, while at 300 s at 125 °C. Similar results were obtained for the other configurations investigated. Therefore, from now on, 125 °C was chosen as the temperature for the fabrication of the µPADs.

Wax spreading through the paper material during heating can be described by Wasburn’s Equation (1), which explains the capillary flow in porous materials [53]:

(1) $$L=\;\sqrt{\frac{\gamma Dt}{4\eta }},$$

where *L* is the distance taken by the fluid in the porous material, *γ* is the surface tension, *D* is the average pore diameter of the porous material, *t* is the time and *η* is the viscosity of the fluid, which is a function of the temperature. In Washburn’s equation, *L* is independent of the width of the printed channel (*W_ci_*). Therefore, the final channel width (*W_cf_*) can be calculated by Equation (2), whereas *W_ci_* is the initial (printed) channel width, and *L* is the distance taken by the fluid.

(2) $$W_{cf}=W_{ci}-2L.$$

Moreover, Carrilho et al. [54] claimed that the Washburn equation is useful when the amount of available wax is a not a limiting factor, which is the case of wax barriers higher than 300 µm widths. On the other hand, Fu and coworkers in 2017 [55] claimed that, during heating, independently of the printed wax barrier width, wax quickly becomes a limiting factor; thus, Washburn equation is not applicable to predict µPADs dimensions. Despite all of the mentioned findings, researchers mainly consider the generation of effective hydrophobic barriers for their paper devices, while the reproducibility on the dimensions of the devices are not fully controlled, reaching the desired design through trial and error. In our case, we aimed to optimize the fabrication process of paper devices by predicting the final dimensions of the device before production. Considering the works from Carrilho and Fu, in our experiments, the fabricated µPADs were designed with wax barriers wider than 300 µm, where the amount of wax is always enough to complete the spreading process.

**Figure 2:**
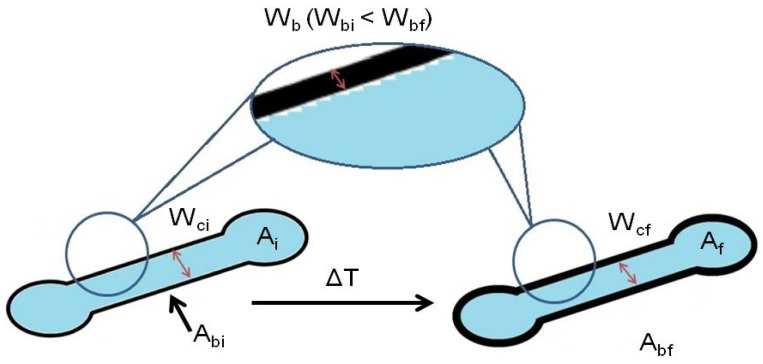
Scheme of a µPAD depicting the parameters before (left) and after (right) the heating step; being *W_ci_* the wax barrier before heating, *A_i_* the internal area before heating and *A_bi_* the wax barrier area before heating; and *W_cf_* the wax barrier after heating, *A_f_* the internal area after heating and *A_bi_* the wax barrier area after heating.

The majority of the works published so far on the understanding of the wax printing behavior for the fabrication of µPADs focused on monitoring the width of the wax barrier and its spreading through the paper. However, none of them investigated the parameters that control the final dimensions of the device. The understanding of these parameters could allow predicting, before the printing step, the final dimensions of the device obtained after the heating step. This will enable obtaining devices acceptable for mass production. To predict the µPAD dimensions, first, the differences between the dimensions of the designed device and the wax printed device have to be taken into account [54]. We found out that the differences between them are always lower than 6% when using the XEROX ColorQube 8580. A slightly different error value could be obtained by using other types of printers. Then, several parameters were considered in order to fabricate reproducible and useful µPADs using the wax printing method (Figure 2). Apart from the heating temperature and heating time characterized above, the initial/final channel width (*W_c_*) and the initial/final width of the wax barrier (*W_b_*), which are commonly used for the determination of µPAD dimensions, as well as the initial/final internal device area (*A*) and the initial/final wax barrier area (*A_b_*) were investigated.

Once the heating process was optimized, and considering that the application of Washburn’s equation did not provide a clear path to predict the dimensions of designed µPADs, we investigated other parameters, such as the internal area of the µPAD (*A*) and the wax barrier area (*A_b_*), instead of just the common *W_b_* and *W_c_.* We thought that the working area (*A_f_*) (final internal area of the µPAD) was an essential parameter for the fabrication of µPADs, since it provides information about the dimensions and the loading capacity of the device (considering the thickness of the substrate). The variables that influence the diffusion of the wax and, consequently, the final internal area were investigated using a series of devices with simple shapes: squares, rectangles and circles. The devices were printed with different internal areas (*A*) and *W_b_,* ranging from 0.50 to 3.5 mm (defining the wax barrier areas, *A_b_*) considering all the wax limitations mentioned before.

The devices were designed, printed and scanned to measure their initial dimensions. Then, they were heated using the optimized conditions established during the previous study (125 °C, 360 s) and scanned again to obtain their final dimensions. The variable shapes, sizes, *W_b_*, *W_c_*, *A* and *A_b_* were analyzed and compared to each other to observe whether the dimensions of the device and/or the *A_b_* affect the final internal area. At the same time, we studied the relation between the initial and the final internal areas, *A*.

**Figure 3:**
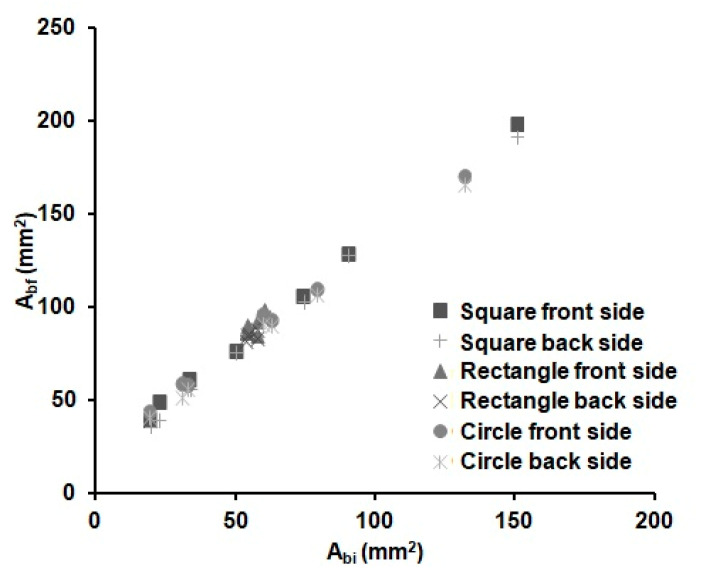
Variations of the front (square, triangle and circle markers) and back (crossed markers) wax barrier areas before (*A_bi_*) and after (*A_bf_*) heating for 6 min at 125 °C of the devices from Table 2 (*n* = 8 per device), with different shapes and barrier width values.

The distance reached by the melted wax (*L*) could be different in the *x* (*L_x_*) and *y* (*L_y_*) dimensions, due to the anisotropy of the paper. However, we found out that the difference in *L_x_* and *L_y_* is negligible, as observed by Carrilho et al. [54] too; see Equation (2). Whereas, since the front and back areas of the substrate were found to be equal within the error, we could deduce that the vertical distance (*L_z_*) filled by the wax is equal to the thickness of the substrate. Figure 3 shows the relationship between the areas of the wax barriers before (*A_bi_*) and after heating (*A_bf_*). There is a linear increase with the width of the wax barrier for all the devices independently of the shape (cycles, square or rectangles) and the dimensions of the device. It can be observed that the *A_bf_* value linearly increases with the width of the barrier. This is an expected result, since there is always enough wax available to spread through the substrate for the investigated time. Besides, it was experimentally observed that it is not possible to distinguish the shape of the device in this figure; therefore, the spreading of the wax seems to be independent of the shape of the device. This fact reduces the number of parameters needed to characterize the wax printing behaviour and, thus, the prediction of the final dimensions of a µPAD.

**Figure 4:**
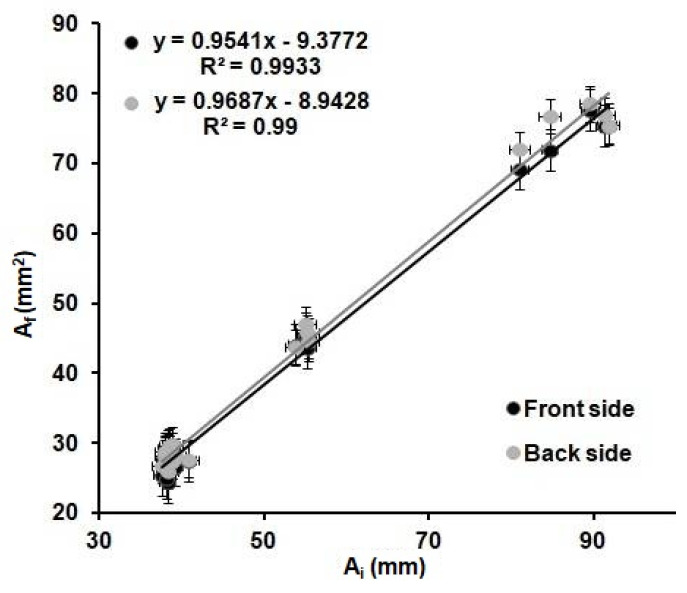
Relation between the internal before heating, *A_i_,* of the printed devices, and the internal area after heating, *A_f_,* of the devices for 6 min at 125 °C; front (black) and back (grey) sides of the devices from Table 2 (*n* = 8 per device).

Figure 4 shows the relation observed between the *A_i_* and the *A_f_* for all the shapes and wax barrier width values. A linear trend is observed when plotting the values obtained from all the devices investigated. It can be determined that the variations of the internal area are the same for all the devices regardless of the shape and width of the wax barrier. The dimensions of the *A_b_* and, so, the *W_b_* do not affect the *A_f_* of the device.

**Figure 5:**
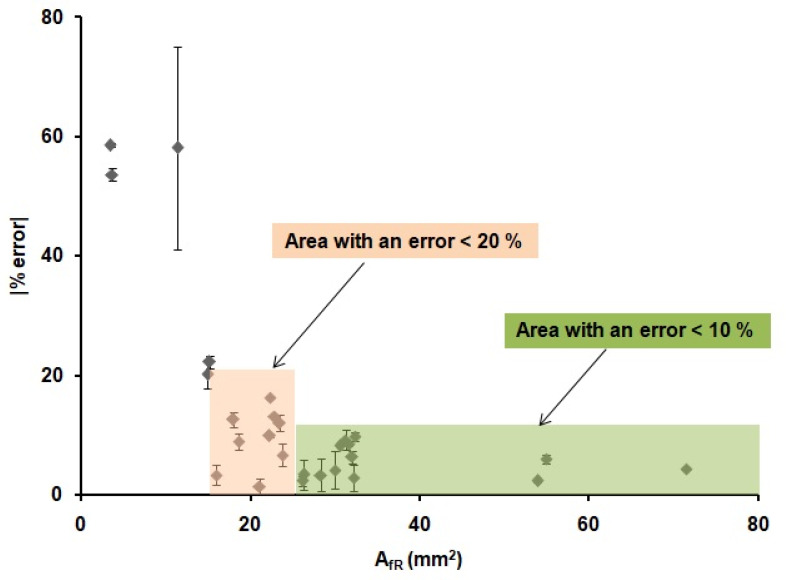
Absolute value of the relative error (%) of the real internal areas (*A_fR_*) of the 26 µPADs for different device configurations obtained when using the equation from Figure 4.

These results demonstrate that the optimization of the printing and heating processes described before are adequate for the fabrication of reproducible devices, since the values of the front and back internal areas are the same within the error, regardless of the shape and width of the printed wax barriers. Moreover, there is a relationship between the areas of the printed devices and the areas of the devices after heating. This relationship could allow us to predict the final dimensions of the µPADs from their original designed dimensions. In order to do that, the linear regression obtained in Figure 4 was used to predict the dimensions of the devices. Twenty-six microfluidic paper-based analytical devices(µPADs)of diverse dimensions and shapes were used as a proof of concept. The internal areas of functional µPADs, after heating, were calculated based on their dimensions, *A_fT_*. Then, these areas were used to calculate the theoretical internal area before heating, *A_iT_*, by means of the plot from Figure 4, *A_fT_* = 0.9687*A_It_*-8.9428. Next, these *A_iT_* values were designed in AutoCAD with different shapes and printed in the paper substrate. The printed devices were first scanned, and their dimensions were measured, using the same method explained above and, then, compared with the dimensions in the drawings. The error generated during the printing process was founded to be ± 6%, as stated before. This error does not affect our study, since all measurements were directly done from the printed devices; thus, this error is already incorporated in the measurements. Later, the devices were heated and scanned again to obtain the real final internal area, *A_fR_*. With these values and the *A_fT_* values, the differences between the designed devices and the real ones can be predicted, and, consequently, the relative error of the prediction of the area can be obtained. The absolute value of the relative error of the predicted areas was studied through Equation (3) and plotted in Figure 5.

(3) $$\left|\varepsilon \%\right|=\left|\frac{A_{fT}-A_{fR}}{A_{fT}}\right|\times 100$$

The results presented in Figure 5 indicate that it is possible to predict the internal area that a µPAD will have after heating from the initial design area. We were able to predict the value of the *A_fR_* with an error lower than a 10% when the areas of the µPADs were higher than 25 mm^2^ (green region in Figure 5) in 12 out of 26 devices. Moreover, an error lower than a 20% was observed when the areas of the µPADs were higher than 15 mm^2^ (red region in Figure 5) in 22 out of 26 devices, independently of the shape of the devices and the width of their wax barriers. For smaller areas, the error was considered too high to be acceptable. The possibility to predict the internal area that a µPAD will have after the printing and heating process allowed us to design devices with known final volumes, since the thickness of the paper is a known value (in this case, 0.18 mm) and with well-defined dimensions. This methodology allows to have a better fluidic control of the µPAD, since the final dimensions of the device are known from the drawing step. These results, combined with the work from Washburn et al. [54], which defined the minimum channel width value needed in a µPAD, complete the requirements for the design and prediction of functional µPADs.

## 4. Conclusions

Nowadays, µPADs is a dynamic area of research with a wide range of applications. Wax printing and heating is the most common µPAD fabrication method, which is used to fabricate large amounts of devices in a very short time. However, this methodology has an inherent lack of control of the fabrication process, so reproducible µPAD features and, thus, fluidic flow control are not possible, being the main obstacles for their mass production and commercialization. Therefore, an unsolved task is to be able to generate uniform microfluidic channels that are reproducible from device to device.

Here, we investigated the heating step of the method, a critical step to generate reproducible devices, since the final dimensions of the µPAD are defined after heating. The use of 125 °C for 6 min generated uniform microfluidic channels with equal width dimensions on both sides. Then, we developed a systematic protocol to monitor the factors that could affect the final dimensions of the device: (1) the shape of the device, (2) wax barrier (*W_bi_*) and (3) the initial internal area of the device or initial channel width (*A_i_*/*W_ci_*). The analysis of the independent variations of those parameters, under the optimized heating conditions, permitted us to predict the final internal area (*A_f_*) and, thus, the final width of the channel (*W_cf_*), considering only the initial internal area (*A_i_*) and the initial channel width (W*_ci_*) values and ignoring the other parameters. In addition, we established a linear relation between the internal area of the printed µPAD and the internal area after heating. This relation allowed us to predict the final area of a µPAD, with errors of less than 10% for areas from 25 mm^2^ and less than 20% for areas from 15 mm^2^, independently of the device dimension and shape. Furthermore, after designing a µPAD, it is possible to predict the final volume (considering the paper thickness) of the device, which has implications in precise fluid handling, the needed sample volume to run the µPAD and, thus, fluidic control.

It is important to point out that these predictions are suitable for a specific type of paper (Whatman Filter paper Grade 1) and printer (XEROX ColorQube 8580), so other papers or printers will require calibrations before use. Nevertheless, this study evidenced a linear relation dependence of the printed internal area of the device and the final internal area after fabrication independently of the shape, *W_bi_* and *A_bi_*, allowing the prediction of the final dimensions of a µPAD. This is an important relationship to consider during mass fabrication, since it allows changing the final dimensions of a device only with the previous calibrations of the printing process.

Our work suggests a method that allows users to predict the final dimensions of the device with a considerable level of accuracy. It reduces costs and saves time during the fabrication of the devices, since it prevents users from having to print and heat countless wax-based paper devices until reaching the desired design through trial and error.

## Figures and Tables

**Table 1:** Design specifications of the microfluidic paper-based analytical devices (µPADs) and picture, *n* = 3; error: ± 0.05.

Name	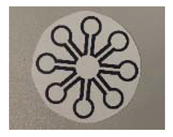 Device-1	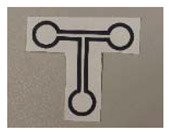 Device-2	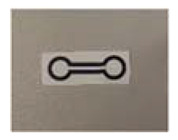 Device-3	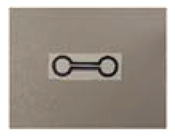 Device-4	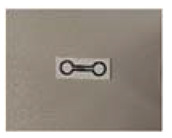 Device-5
**Channel width (mm)**	1.50	1.50	2.25	1.50	1.00
**Barrier width (mm)**	2.25	2.25	3.38	2.25	1.50
**Channel length (mm)**	12	24	36	24	16
**Diameter inlet/outlet (mm)**	14 and 12	12	18	12	8

**Table 2:** Designspecifications of the µPADs.

**Square**				** 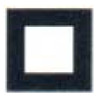 **					
**Barrier width (mm)**	0.60		0.99	1.42	1.96	2.52	0.58		2.99
**Internal area (mm^2^)**	55		55	55	55	40	85		90
**Rectangle**				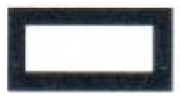					
**Barrier width (mm)**	0.85	0.87	0.83	1.68	1.71	1.74	1.63	1.66	1.81
**Internal area (mm^2^)**	79	77	77	38	38	38	39	39	38
**x (mm)**	13.10	12.60	16.90	10.30	8.50	9.40	7.70	6.80	5.80
**y (mm)**	6.03	6.11	4.56	3.69	4.47	4.04	5.06	5.74	6.55
**Circle**				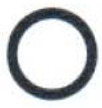					
**Barrier width (mm)**	0.88		1.54	3.02	0.77	1.26	2.17		2.61
**Internal area (mm^2^)**	81		90	92	39	38	38		38

**Table 3:** Time required for the fabrication of µPADs with the same shapes at the top and at the bottom sides of the paper substrate at two temperatures, *n* = 3; error: ±5 s.

	Time (s)	
	110 °C	125 °C
**Device-1**	540	420
**Device-2**	540	360
**Device-3**	480	420
**Device-4**	540	420
**Device-5**	420	300

## Data Availability

Data available on request.
